# *Codium fragile* Suppresses PM_2.5_-Induced Cognitive Dysfunction by Regulating Gut–Brain Axis via TLR-4/MyD88 Pathway

**DOI:** 10.3390/ijms241612898

**Published:** 2023-08-17

**Authors:** Tae Yoon Kim, Jong Min Kim, Hyo Lim Lee, Min Ji Go, Seung Gyum Joo, Ju Hui Kim, Han Su Lee, Dong Yeol Lee, Hyun-Jin Kim, Ho Jin Heo

**Affiliations:** 1 Division of Applied Life Science (BK21), Institute of Agriculture and Life Science, Gyeongsang National University, Jinju 52828, Republic of Korea; kty8747@naver.com (T.Y.K.); myrock201@gnu.ac.kr (J.M.K.); gyfla059@gnu.ac.kr (H.L.L.); rh9245@naver.com (M.J.G.); s716q@naver.com (S.G.J.); zkfkapflove@nate.com (J.H.K.); ns3005@naver.com (H.S.L.); hyunjkim@gnu.ac.kr (H.-J.K.); 2 Research & Development Team, Gyeongnam Anti-Aging Research Institute, Sancheong 52215, Republic of Korea; dylee1984@gari.or.kr

**Keywords:** *Codium fragile*, particulate matter, gut–brain axis

## Abstract

This study was conducted to evaluate the cognitive dysfunction improvement effect of aqueous extract of *Codium fragile* (AECF) by regulating the imbalance of the gut–brain axis in chronic particulate matter (PM)_2.5_-exposed mice. The physiological compounds of AECF were identified as hexadecanamide, oleamide, octadecanamide, stearidonic acid, and linolenic acid by the ultra-performance liquid chromatography-quadrupole time of flight mass spectrometry (UPLC Q-TOF MS^E^) analysis. To evaluate the effect of PM_2.5_ on the antioxidant system, superoxide dismutase (SOD) contents, reduced glutathione (GSH) contents, and malondialdehyde (MDA) contents were measured in colon and brain tissues. AECF significantly ameliorated the imbalance of the antioxidant systems. Also, AECF improved intestinal myeloperoxidase (MPO) activity, the abundance of the gut microbiome, short-chain fatty acids (SCFAs) contents, and tight junction protein expression against PM_2.5_-induced damage. In addition, AECF prevented PM_2.5_-induced inflammatory and apoptotic expression via the toll-like receptor-4 (TLR-4)/myeloid differentiation primary response 88 (MyD88) pathway in colon and brain tissues. Additionally, AECF enhanced the mitochondrial function, including the mitochondrial membrane potential (MMP) and reactive oxygen species (ROS) contents in brain tissues. Furthermore, AECF regulated the cholinergic system, such as acetylcholine (ACh) contents, acetylcholinesterase (AChE) activity, and protein expression levels of AChE and choline acetyltransferase (ChAT) in brain tissues. To evaluate the effect of cognitive dysfunction caused by PM_2.5_-induced intestinal dysfunction, behavior tests such as Y-maze, passive avoidance, and Morris water maze tests were performed. From the results of the behavior tests, AECF ameliorated spatial learning and memory, short-term memory, and long-term learning and memory function. This study confirmed that AECF reduced PM_2.5_-induced cognitive dysfunction by regulating gut microbiome and inflammation, apoptosis, and mitochondrial function by enhancing the gut–brain axis. Based on these results, this study suggests that AECF, which contains fatty acid amides, might be a potential material for ameliorating PM_2.5_-induced cognitive dysfunction via gut–brain axis improvement.

## 1. Introduction

Air pollution is one of the biggest environmental problems facing humans in the 21st century, and the level of air pollution is getting worse globally due to rapid economic development and industrialization [1]. One of the most serious causes of air pollution in recent years is particulate matter (PM), which consists of heavy metals, carbon monoxide, sulfates, nitrates, and polycyclic aromatic hydrocarbons (PAHs) that are harmful to the human body [2]. Particulate matter is classified into PM_10_ (≤10 μm) and PM_2.5_ (≤2.5 μm) according to its diameter, and because of its small diameter, it penetrates the human body and circulates throughout the body, causing various diseases in the human body [3]. Inhaled PM_2.5_ can easily pass through blood vessels and circulate throughout the whole body to reach several organs, causing apoptosis and inflammation with the activation of the toll-like receptor-4 (TLR-4)/myeloid differentiation primary response 88 (MyD88) pathway [4]. In addition, reactive oxygen species (ROS), oxidative stress, and mitochondrial dysfunction caused by exposure to fine dust cause various diseases in the human body [5]. Additionally, PM_2.5_ reaches the gut through pulmonary mucus transport or direct intake; PM_2.5_ that enters the gut can cause inflammation by increasing the expression of cytokines and changing the gut microbiome [6]. The gut microbiome is a group of microorganisms that perform various functions such as polysaccharide digestion, immune system development, infection prevention, fat storage, vitamin synthesis, angiogenesis, and behavioral development [7]. Changes in the gut microbiome are known to affect the brain through the gut–brain axis, a communication network between the gut and brain [8]. The gut–brain axis regulates numerous brain functions, with the gut microbiome modulating blood–brain barrier formation and integrity, microglia maturation and ramification, neurotrophins, neurotransmitters, and neurogenesis. In a recent study, it was found that imbalance of gut microbiome and neurodegenerative diseases are closely related through the gut–brain axis [9]. Furthermore, these changes in the gut microbiome cause neuronal inflammation and apoptosis through dysfunction of the gut–brain axis, causing cognitive impairment such as that found in Alzheimer’s disease (AD) [10,11]. Absorption of PM_2.5_ changes the abundance of the gut microbiome and the content of kynurenine metabolites that act as neuronal protection in the gut–brain axis [12]. Ultimately, damage to the gut–brain axis caused by PM_2.5_ can further worsen cognitive dysfunction. Therefore, it is necessary to enhance the gut environment with the intake of natural products that improve the gut-brain axis to prevent cognitive impairment.

*Codium fragile* is a marine green alga belonging to the family *Codiaceae* and ranges from 10 to 40 cm high and includes repeatedly branching cylindrical segments [13]. It has been used for food in Korea for a long time and is also used for food in China and Japan [14]. *Codium fragile* contains a variety of bioactive compounds such as *p*-coumaric acid, gallic acid, 4-hydroxybenzoic acid, 4-hydroxybenzaldehyde, salicylic acid, biochanin A, and diosgenin [13]. In recent studies, *Codium fragile* has been reported to have physiological activities such as anti-obesity as a probiotic, inhibition of food edema inflammation, and skin improvement [15,16,17]. In addition, *Codium fragile* ethanol extract had an anti-inflammatory effect in LPS-induced RAW 264.7 cells [18]. However, there is little research on *Codium fragile* between PM_2.5_ and the gut–brain axis. In a previous study, the protective effect of aqueous extract of *Codium fragile* on brain and respiratory tissues with PM-induced cytotoxicity was confirmed [19]. Therefore, this study was conducted to confirm the improvement effect of aqueous extract of *Codium fragile* on the gut–brain axis and cognitive function induced by PM_2.5_ cytotoxicity using a PM-exposure BALB/c mice model.

## 2. Results

### 2.1. Identification of Compound Using UPLC-QTOF/MS^E^

**Figure 1:**
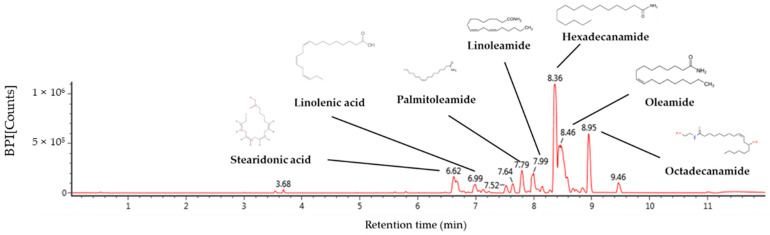
UPLC Q-TOF/MS^E^ chromatographic spectra in positive ion mode.

The physiological compounds of the aqueous extract of the *Codium fragile* (AECF) hexane layer were qualitatively identified using ultra-performance liquid chromatography-quadrupole time of flight mass spectrometry (UPLC-QTOF/MS^E^) analysis (Figure 1 and Table 1). Identified compounds were confirmed by comparison with MS fragments from previous studies. Stearidonic acid (6.62 min, *m*/*z* 277; fragmentation, 107, 93, 91, 79, and 67), linolenic acid (6.99 min, *m*/*z* 279; fragmentation, 227, 157, 95, and 81), palmitoleamide (7.79 min, *m*/*z* 254; fragmentation, 237, 219, 149, and 135), linoleamide (7.99 min, *m*/*z* 280; fragmentation, 263, 245, 175, 113, 95, and 81), hexadecanamide (8.36 min, *m*/*z* 256; fragmentation, 116, 102, 88, 74, and 57), oleamide (8.46 min, *m*/*z* 282; fragmentation, 283, 265, 247, 240), and octadecanamide (8.95 min, *m*/*z* 284; fragmentation, 285, 102, and 88) were detected as compounds.

### 2.2. Saccharide and Sulfate Analysis

The total polysaccharide and sulfate contents are shown in Table 2. Total polysaccharide contents of AECF were measured as 31.81%, and total sulfate contents were measured as 31.05%. In the results of monosaccharide composition, galactose (48.81%) was the highest content, followed by arabinose (21.35%), glucose (20.27%), xylose (7.84%), fucose (0.89%), and rhamnose (0.80%).

### 2.3. Antioxidant System Test

#### 2.3.1. Superoxide Dismutase (SOD) Contents

**Figure 2:**
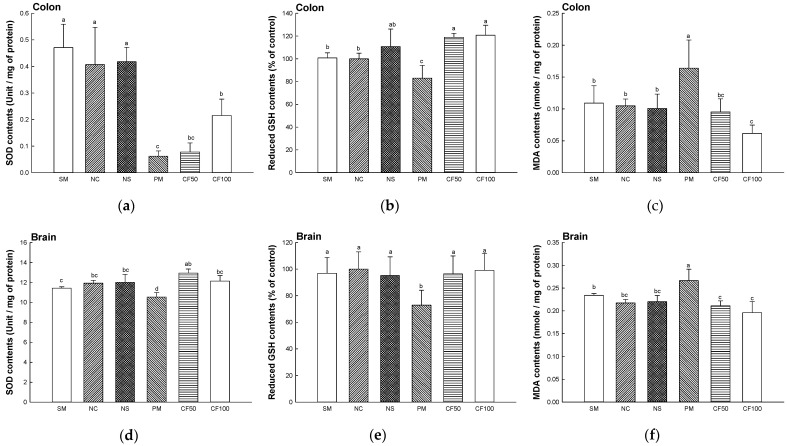
Protective effect of AECF on superoxide dismutase (SOD) activity, reduced glutathione (GSH) contents and malondialdehyde (MDA) contents in colon ((**a**–**c**)) and brain tissue ((**d**–**f**)). Results shown are mean ± SD (*n* = 5). Data were statistically considered at *p* < 0.05, and different small letters represent statistical differences.

The SOD contents in the colon and brain tissues were shown in Figure 2a,d. The SOD activity of the SM (colon, 0.47 unit/mg of protein; brain, 11.42 unit/mg of protein), NC (colon, 0.40 unit/mg of protein; brain, 11.92 unit/mg of protein), and NS (colon, 0.41 unit/mg of protein; brain, 12.00 unit/mg of protein) groups represented no significant differences. The SOD contents of the PM group (colon, 0.06 unit/mg of protein; brain, 10.53 unit/mg of protein) were decreased compared to the NC group. However, SOD contents of the CF50 (colon, 0.07 unit/mg of protein; brain, 12.93 unit/mg of protein) and CF100 (colon, 0.21 unit/mg of protein; brain, 12.12 unit/mg of protein) groups were increased compared to the PM group.

#### 2.3.2. Reduced Glutathione (GSH) Contents

The reduced GSH contents in the colon and brain tissues are shown in Figure 2b,e. The reduced GSH contents of the SM (colon, 100.80% of control; brain 96.87% of the control), NC (colon, 100% of control; brain, 100% of control), and NS (colon, 110.75% of the control; brain, 95.17% of the control) groups represented no significant differences. The PM (colon, 83.05% of control; brain 72.88% of the control) group showed significantly decreased reduced GSH contents. However, the reduced GSH contents of CF50 (colon, 118.92% of control; brain 96.48% of control) and CF100 (colon, 120.74% of control; brain, 99.02% of control) groups were significantly increased compared to the PM group.

#### 2.3.3. Malondialdehyde (MDA) Contents

The MDA contents in the colon and brain tissues are shown in Figure 2c,f. The MDA contents of the SM (colon, 0.10 nmole/mg of protein; brain, 0.23 nmole/mg of protein), NC (colon, 0.10 nmole/mg of protein; brain, 0.21 nmole/mg of protein), and NS (colon, 0.10 nmole/mg of protein; brain, 0.22 nmole/mg of protein) groups represented no significant differences. The PM (colon, 0.16 nmole/mg of protein; brain 0.26 nmole/mg of protein) group showed significantly increased MDA contents. However, MDA contents of the CF50 (colon, 0.09 nmole/mg of protein; brain 0.21 nmole/mg of protein) and CF100 (colon, 0.06 nmole/mg of protein; brain 0.19 nmole/mg of protein) groups were significantly decreased compared to the PM group.

#### 2.3.4. Myeloperoxidase (MPO) Activity

**Figure 3:**
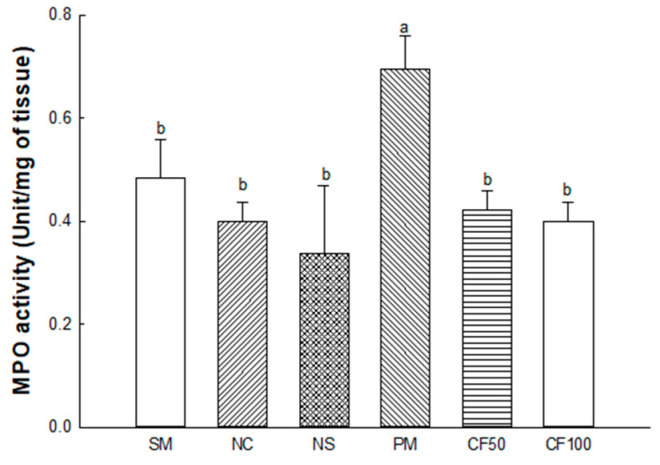
Protective effect of AECF on myeloperoxidase (MPO) activity in colon. Results shown are mean ± SD (*n* = 3). Data were statistically considered at *p* < 0.05, and different small letters represent statistical differences.

The MPO activity in the colon tissues is shown in Figure 3. The MPO contents of the SM (0.48 Unit/mg of tissue), NC (0.40 Unit/mg of tissue), and NS (0.33 Unit/mg of tissue) groups represented no significant differences. The PM (0.69 Unit/mg of tissue) group showed significantly increased MDA contents. However, the MDA contents of the CF50 (0.42 Unit/mg of tissue) and CF100 (0.40 Unit/mg of tissue) groups were significantly decreased compared to the PM group.

### 2.4. The Regulation Effect of AECF on Gut Microbiome Abundance

**Figure 4:**
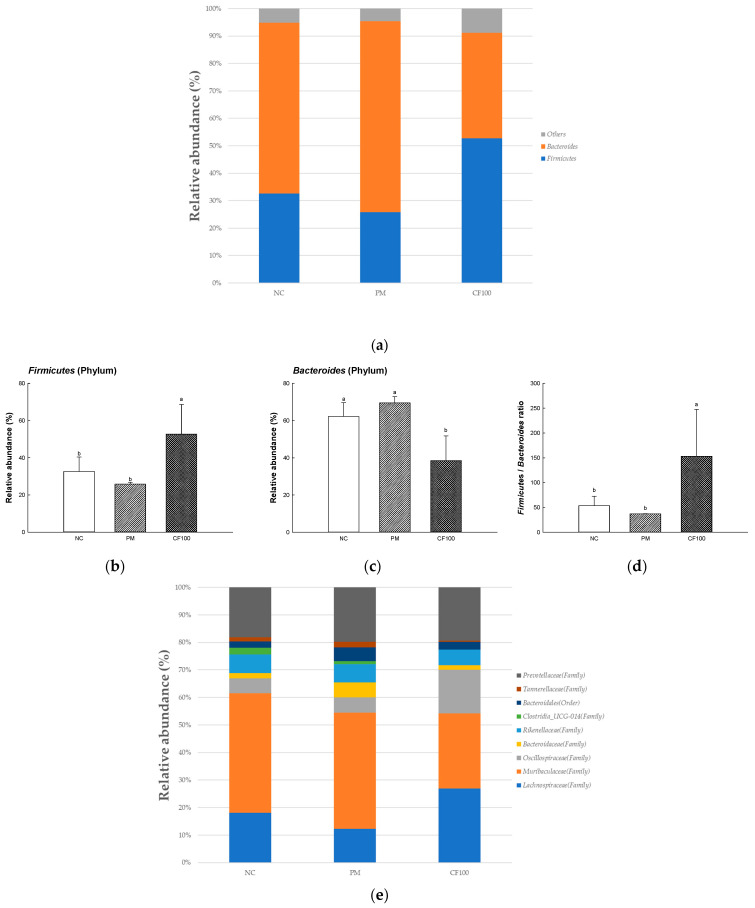

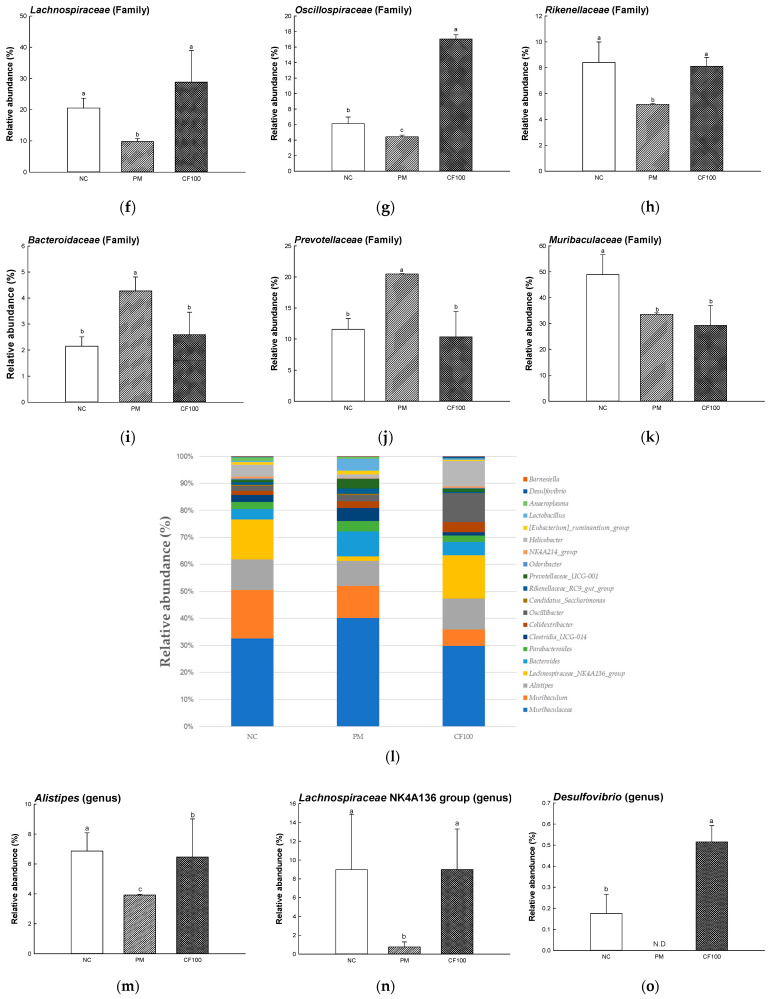

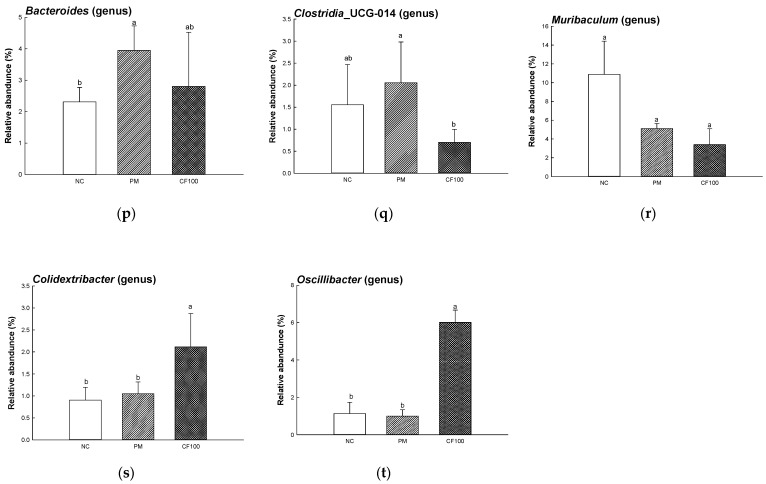
The variation in the gut microbiome by AECF in PM_2.5_-induced gut dysbiosis. The relative abundance of phylum (**a**), *Firmicutes* (**b**), *Bacteroides* (**c**), *Firmicutes*/*Bacteroides* (**d**), relative abundance of family (**e**), *Lachnospiraceae* (**f**), *Oscillospiraceae* (**g**), *Rikenellaceae* (**h**), *Bacteroidaceae* (**i**), *Prevotellaceae* (**j**), *Muribaculaceae* (**k**), relative abundance of genus (**l**), *Alistipes* (**m**), *Lachnospiraceae* NK4A136 group (**n**), *Desulfovibrio* (**o**), *Bacteroides* (**p**), *Clostridia*_UCG-014 (**q**), *Muribaculum* (**r**), *Colidextribacter* (**s**), and *Oscilibacter* (**t**). Results shown are mean ± SD (*n* = 3). Data were statistically considered at *p* < 0.05, and different small letters represent statistical differences.

To evaluate the regulation effect of AECF on gut microbiome, the relative abundances were measured (Figure 4). At the phylum level, comparing the abundance of *Firmicutes* and *Bacteroides* in the NC (32.60% and 62.20%, respectively) and PM (25.82% and 69.49%, respectively) groups, the CF100 group significantly increased the abundance of *Firmicutes* (52.72%) and significantly decreased the abundance of *Bacteroides* (38.50%). At the family level, the abundances of *Lachnospiraceae* (9.78%), *Oscillospiraceae* (4.43%), and *Rikenellaceae* (5.16%) of the PM group were decreased compared with the NC (20.52%, 6.11%, and 8.40%, respectively) group, and the CF100 (28.82%, 17.03%, and 8.10%) group showed improved abundance. In addition, the abundances of *Bacteroidaceae* (4.27%) and *Prevotellaceae* (20.48%) of the PM group were increased compared with the NC (2.14% and 11.53%, respectively) group, and the CF100 (2.59% and 10.34%, respectively) group showed decreased abundance. Compared with the NC group (48.10%), the abundances of *Muribaculaceae* of the PM group (33.53%) and CF100 group (29.22) were decreased. At the genus level, in the PM group, the abundance of *Alistipes* (3.92%), *Lachnospiraceae* NK4A136 group (0.75%), and *Desulfovibrio* (not detected) were decreased compared with the NC group, (6.86%, 8.97%, and 0.17%, respectively) and the CF100 (6.47%, 8.98%, and 0.51%, respectively) group showed improved abundance. In the PM group, the abundances of *Bacteroides* (3.94%), and *Clostridia_UCG-014* (2.05%) were increased compared with the NC group (2.31%, and 1.55%, respectively), and the CF100 group (2.80%, and 0.69%, respectively) showed decreased abundance. Compared with the NC group (10.88%), the abundance of *Muribaculum* showed no significant difference in the PM group (5.1%) and the CF100 group (3.39%). Compared with the NC group (0.90% and 1.13%, respectively) and the PM group (1.05% and 0.99%, respectively), the abundances of *Colidextribacter* and *Oscilibacter* were increased in the CF100 group (2.11% and 6.00%, respectively).

### 2.5. The Regulation Effect of AECF on SCFA Contents

**Figure 5:**
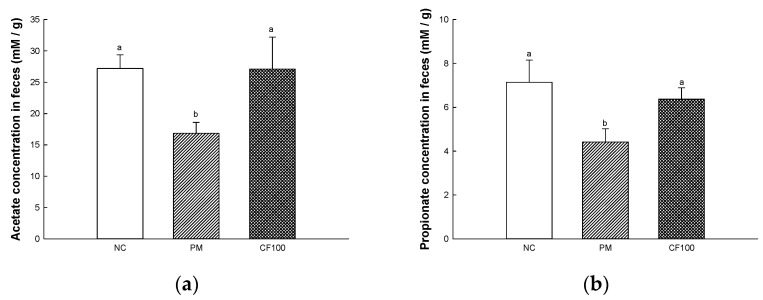
The short chain fatty acid (SCFA) contents in mice feces. Results shown are mean ± SD (*n* = 5). The acetate concentration (**a**) and propionate concentration (**b**) in feces. Data were statistically considered at *p* < 0.05, and different small letters represent statistical differences.

The SCFAs concentration in the feces are shown in Figure 5. The fecal SCFAs concentrations of the PM group (acetate, 16.86 mM/g; propionate, 4.41 mM/g) were significantly downregulated compared to the NC group (acetate, 27.17 mM/g; mM/g; propionate, 7.14 mM/g). The CF100 group (acetate, 27.07 mM/g; mM/g; propionate, 6.37 mM/g) improved fecal SCFAs concentration compared to the PM group.

### 2.6. The Regulation Effect of AECF on PM_2.5_-Induced Gut Barrier Dysfunction

**Figure 6:**
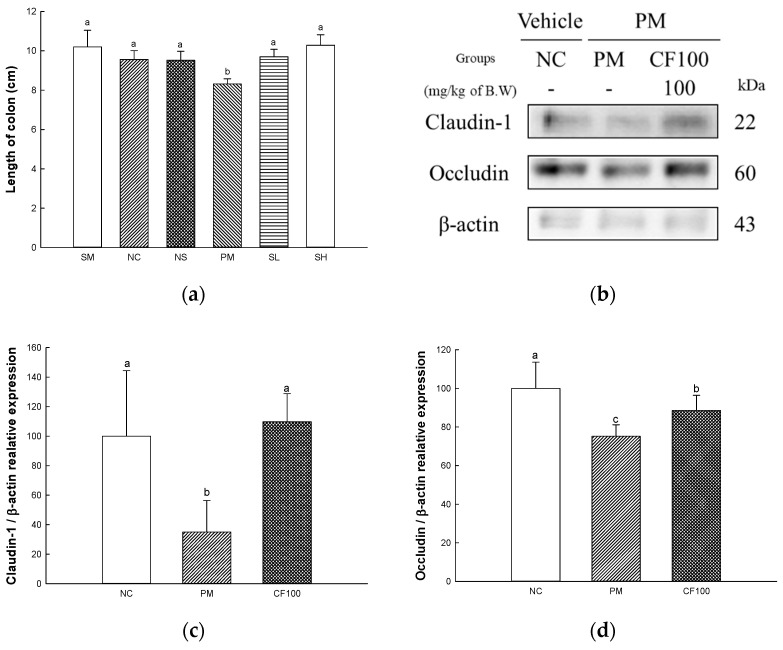
The regulation of AECF on PM_2.5_-induced gut permeability dysfunction. The length of colon (**a**)**,** band images of Western blot analysis (**b**), protein expression levels of claudin-1 (**c**), occludin (**d**) in colon tissues. Results shown are mean ± SD (*n* = 3). Data were statistically considered at *p* < 0.05, and different small letters represent statistical differences.

The length of the colon and the protein expression of claudin-1 and occludin are presented in Figure 6. The gut length is shown in Figure 6a. The gut lengths of the SM (10.20 cm), NC (9.56 cm), and NS (9.52 cm) groups represented no significant differences. The gut length (8.32 cm) of the PM group showed decreased gut length. However, the gut lengths of the CF50 (9.70 cm) and CF100 (10.28 cm) groups were significantly increased compared to the PM group. The protein expression levels of claudin-1 (34.96% of the NC group) and occludin (75.27% of the NC group) of the PM group were significantly downregulated compared to the NC group (Figure 6b–d). The CF100 group upregulated claudin-1 (109.75% of NC group) and occludin (88.60% of NC group) expression levels compared to the PM group.

### 2.7. The Regulation Effect of AECF on PM_2.5_-Induced TLR-4/MyD88 Signaling Inflammation in Colon Tissues

**Figure 7:**
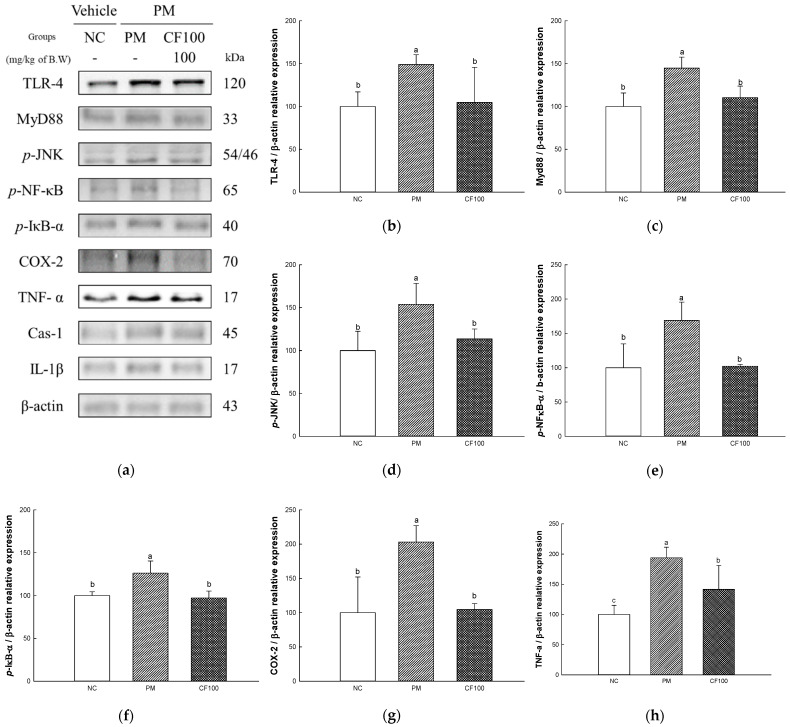

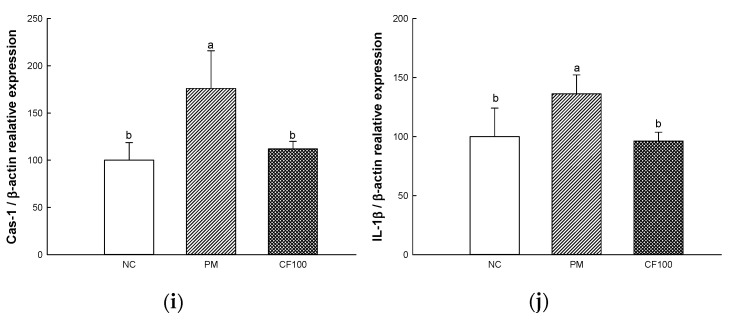
The regulation of AECF on PM_2.5_-induced TLR-4/MyD88 signaling inflammation in colon tissues. Band images of Western blot analysis (**a**), protein expression levels of TLR-4 (**b**), MyD88 (**c**), *p*-JNK (**d**), *p*-NF-κB (**e**), *p*-IκB-α (**f**), COX-2 (**g**), TNF-α (**h**), Cas-1 (**i**), and IL-1β (**j**) in colon tissues. Results shown are mean ± SD (*n* = 3). Data were statistically considered at *p* < 0.05, and different small letters represent statistical differences.

The inflammation protein expression levels TLR-4, MyD88, phosphorylated c-Jun N-terminal kinases (*p*-JNK), phosphorylated nuclear factor kappa-light-chain-enhancer of activated B (*p*-NF-κB), phosphorylated nuclear factor of kappa light polypeptide gene enhancer in B-cell inhibitor alpha (*p*-IκB-α), cyclooxygenase-2 (COX-2), tumor necrosis factor alpha (TNF-α), caspase-1 (Cas-1), and interleukin (IL)-1β were measured in colon tissues (Figure 7). The expression levels of TLR-4 (148.98% of NC group), MyD88 (144.82% of NC group), *p*-JNK (153.86% of NC group), *p*-NF-κB (168.87% of NC group), *p*-IκB-a (126.18% of NC group), COX-2 (203.09% of NC group), TNF-α (193.76% of NC group) Cas-1 (176.00% of NC group), and IL-1β (136.23% of NC group) of the PM group were significantly upregulated to the NC group. The administration of CF100 downregulated TLR-4 (104.75% of NC group), MyD88 (110.35% of NC group), *p*-JNK (113.85% of NC group), *p*-NF-κB (102.25% of NC group), *p*-IκB-α (97.25% of NC group), COX-2 (104.67% of NC group), TNF-α (141.74% of NC group), Cas-1 (112.01% of NC group), and IL-1β (96.34% of NC group) expression levels compared to the PM group.

### 2.8. The Regulation Effect of AECF on PM_2.5_-Induced Apoptosis in Colon

**Figure 8:**
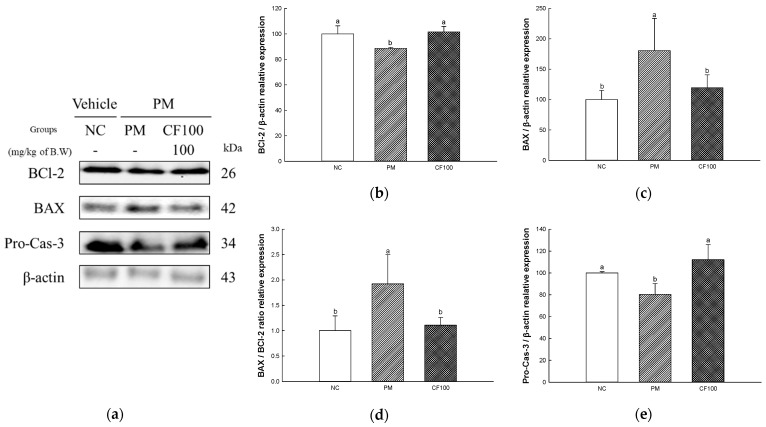
The regulation of AECF on PM_2.5_-induced oxidative stress inflammation in colon tissues. Band images of Western blot analysis (**a**), protein expression levels of BCl-2 (**b**), BAX (**c**), BAX/BCl-2 ratio (**d**), and Pro-Cas-3 (**e**) in colon tissues. Results shown are mean ± SD (*n* = 3). Data were statistically considered at *p* < 0.05, and different small letters represent statistical differences.

The apoptotic protein expressions of B-cell lymphoma 2 (BCl-2), BCl-2-like protein (BAX), and Pro-Cas-3 were measured in colon tissues (Figure 8). The expression levels of BAX (180.43% of NC group, Cas-1 (176.00% of NC group) of the PM group were significantly upregulated compared to the NC group, whereas the CF100 group downregulated BAX (119.56% of NC group) expression levels compared to the PM group (Figure 7c,e,f). The expression levels of BCl-2 (88.60% of NC group) and Pro-Cas-3 (80.40% of NC group) in the PM group were significantly downregulated compared to NC. However, the CF 100 group upregulated BCl-2 (101.56% of NC group) and Pro-Cas-3 (112.11% of NC group) expression levels compared to the PM group.

### 2.9. The Regulation Effect of AECF on PM_2.5_-Induced TLR-4/MyD88 Signaling Inflammation in Brain Tissues

**Figure 9:**
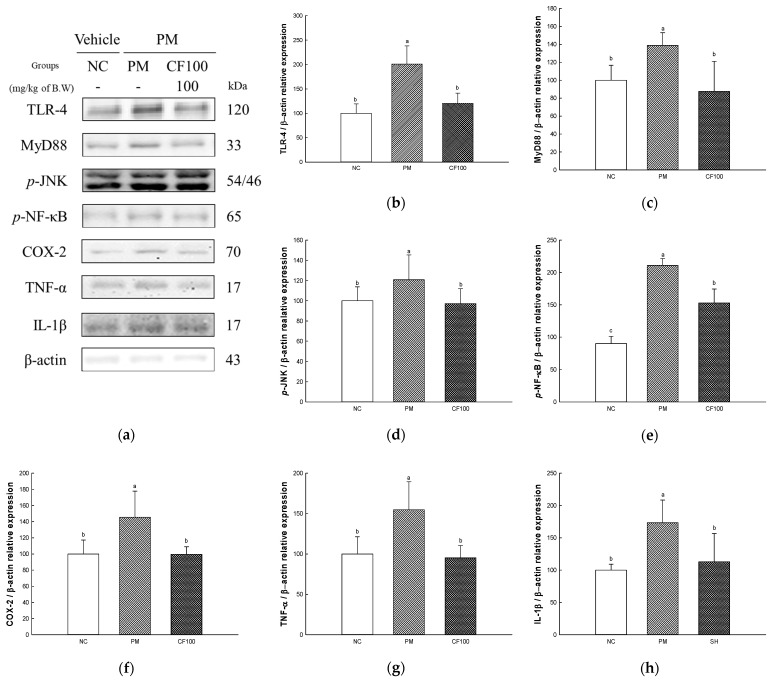
The regulation of AECF on PM_2.5_-induced TLR-4/MyD88 signaling inflammation in brain tissues. Band images of Western blot analysis (**a**), protein expression levels of TLR-4 (**b**), MyD88 (**c**), *p*-JNK (**d**), *p*-NF-κB (**e**), COX-2 (**f**), TNF-α (**g**), and IL-1β (**h**) in brain tissues. Results shown are mean ± SD (*n* = 3). Data were statistically considered at *p* < 0.05, and different small letters represent statistical differences.

The inflammation protein expression levels of TLR-4, MyD88, *p*-JNK, *p*-NF-κB, COX-2, TNF-α), and IL-1β were measured in brain tissues (Figure 9). The expression levels of TLR-4 (200.91% of NC group), MyD88 (138.79% of NC group), *p*-JNK (120.80% of NC group), *p*-NF-κB (234.62% of NC group), COX-2 (145.55% of NC group), TNF-α (154.68% of NC group), and IL-1β (173.28% of NC group) of the PM group were significantly upregulated compared to the NC group. The administration of CF100 downregulated TLR-4 (120.57% of NC group), MyD88 (87.55% of NC group), *p*-JNK (97.10% of NC group), *p*-NF-κB (170.02% of NC group), COX-2 (99.59% of NC group), TNF-α (95.33% of NC group), and IL-1β (112.97% of NC group) expression levels to the PM group.

### 2.10. The Regulation Effect of AECF on PM_2.5_-Induced Apoptosis in Brain

**Figure 10:**
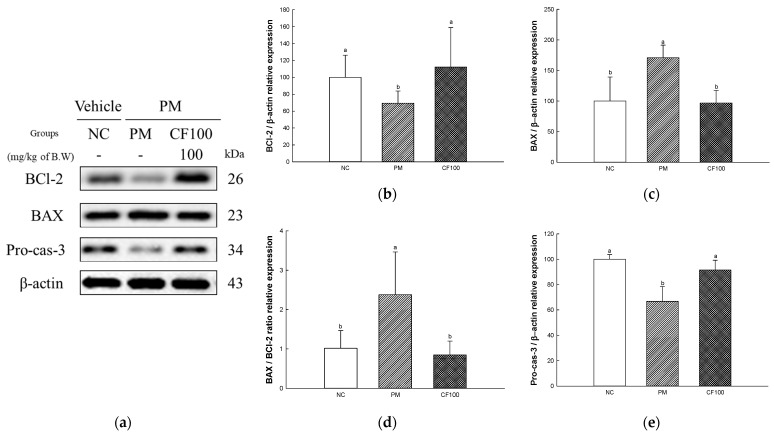
The regulation of AECF on PM_2.5_-induced oxidative stress inflammation in brain tissues. Band images of Western blot analysis (**a**), protein expression levels of BCl-2 (**b**), BAX (**c**), BAX/BCl-2 ratio (**d**) and Pro-Cas-3 (**e**) in brain tissues. Results shown are mean ± SD (*n* = 3). Data were statistically considered at *p* < 0.05, and different small letters represent statistical differences.

The apoptotic protein expressions of BCl-2, BAX, and Pro-Cas-3 were measured in brain tissues (Figure 10). The expression levels of BAX (170.87% of NC group) of the PM group were significantly upregulated compared to the NC group, whereas the CF100 group downregulated BAX (96.90% of NC group) expression levels compared to the PM group (Figure 7c,e,f). The expression levels of BCl-2 (69.30% of NC group), and Pro-Cas-3 (66.75% of NC group) of the PM group were significantly downregulated compared to the NC group. However, the CF 100 group upregulated BCl-2 (112.37% of NC group) and Pro-Cas-3 (91.43% of NC group) expression levels compared to the PM group.

### 2.11. Mitochondrial Activity

#### 2.11.1. Mitochondrial ROS Levels

**Figure 11:**
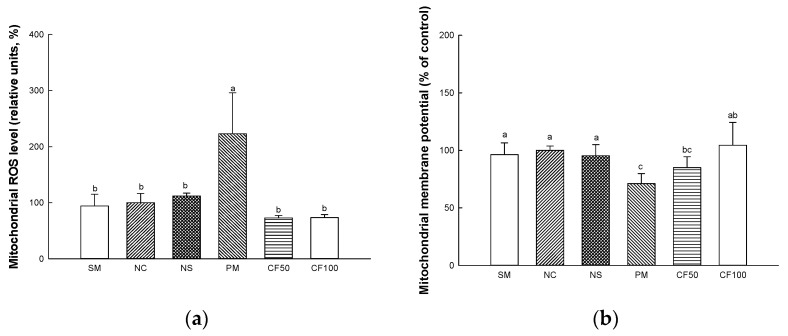
Protective effect of AECF mitochondrial DCF fluorescence (**a**), mitochondrial membrane potential (MMP) (**b**) in brain tissue. Results shown are mean ± SD (*n* = 5). Data were statistically considered at *p* < 0.05, and different small letters represent statistical differences.

The mitochondrial ROS levels in brain tissue are shown in Figure 11a. The mitochondrial ROS levels on brain tissue in the SM (94.17% of control), NC (100% of control), and NS (111.93% of control) groups represented no significant differences. However, mitochondrial ROS (223.12% of control) of the PM group showed significantly increased mitochondrial ROS levels. However, the CF50 (72.84% of control) and CF100 (73.46% of control) groups showed improved mitochondrial ROS levels in the brain.

#### 2.11.2. Mitochondrial Membrane Potential (MMP) Levels

The MMP levels in brain tissues are shown in Figure 11b. The mitochondrial ROS levels on brain tissues in the SM (96.34% of control), NC (100% of control), and NS (95.37% of control) groups represented no significant differences. However, the mitochondrial MMP levels (71.11% of control) of the PM group significantly decreased mitochondrial MMP levels. However, the CF50 (84.99% of control) and CF100 (104.43% of control) groups showed improved mitochondrial MMP levels in brain tissues.

### 2.12. Cholinergic System

#### 2.12.1. Acetylcholine (ACh) Contents

**Figure 12:**
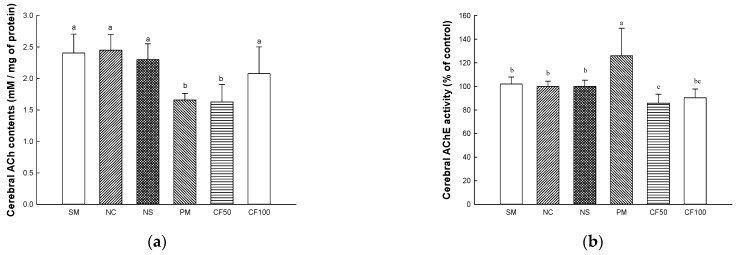

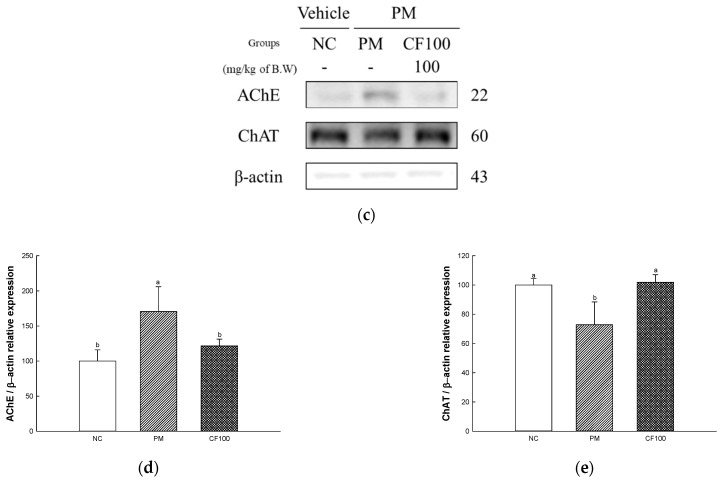
Protective effect of AECF on acetylcholine (ACh) contents (**a**) and acetylcholinesterase (AChE) activity (**b**) in brain tissues, band images of Western blot analysis (**c**), and protein expression levels of AChE (**d**) and choline acetyltransferase (ChAT) (**e**). Results shown are mean ± SD (*n* = 5) in ACh contents and AChE activity, and shown are mean ± SD (*n* = 3) protein expression levels of AChE and ChAT. Data were statistically considered at *p* < 0.05, and different small letters represent statistical differences.

The ACh contents are shown in Figure 12a. The ACh contents on brain tissues in the SM (2.40 mmole/mg of protein), NC (2.44 mmole/mg of protein), and NS (2.30 mmole/mg of protein) groups represented no significant differences. However, the ACh contents (1.65 mmole/mg of protein) of the PM group showed decreased ACh contents. CF50 (1.62 mmole/mg of protein) had no significant differences compared to the PM group. However, the CF100 (2.07 mmole/mg of protein) group was significantly restored compared to the ACh contents of the PM group.

#### 2.12.2. Acetylcholinesterase (AChE) Activity

The AChE activities are shown in Figure 12b. The AChE activities on brain tissues in the SM (102.05% of control), NC (100.00% of control), and NS (100.05% of control) groups represented no significant differences. The AChE activity (125.87% of control) of the PM group showed increased AChE activity. However, the CF50 (85.80% of control) and CF100 (90.38% of control) group were significantly decreased AChE compared to the PM group.

#### 2.12.3. Protein Expression Levels of Cholinergic System

The cholinergic system protein expression levels of AChE and ChAT were measured (Figure 12c–e). The expression levels of AChE (170.62% of NC group) of the PM group were significantly upregulated compared to the NC group, whereas the CF100 group (121.53% of NC group) downregulated expression levels compared to the PM group. The expression levels of ChAT (72.83% of NC group) of the PM group were significantly downregulated compared to the NC group. However, the CF 100 group upregulated ChAT (101.85% of NC group) expression level compared to the PM group.

### 2.13. Behavior Test

#### 2.13.1. Y-Maze Test

**Figure 13:**
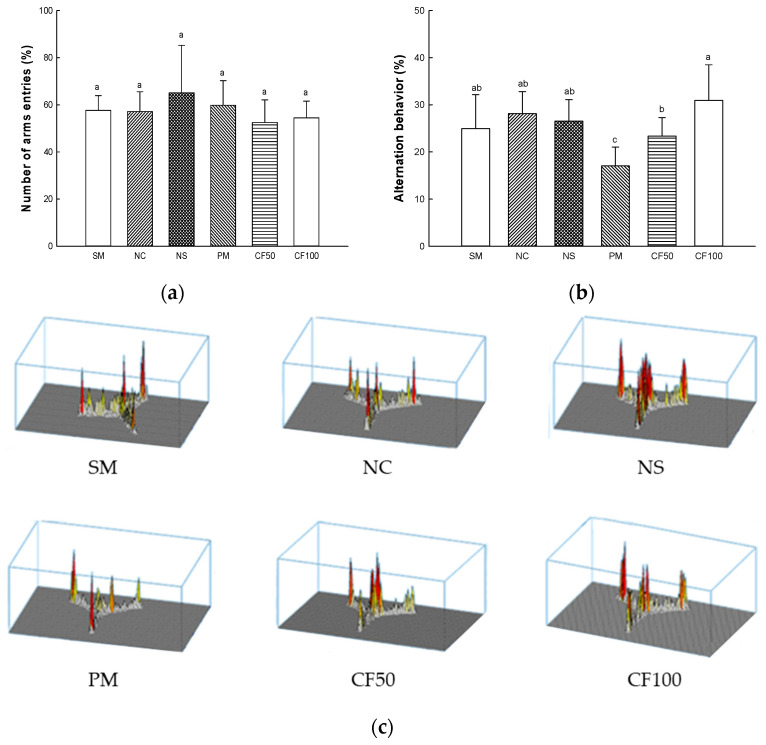
Protective effect of AECF on number of arm entries (**a**), alternation behavior (**b**), and path tracing (**c**) in Y-maze test. Results shown are mean ± SD (*n* = 5). Data were statistically considered at *p* < 0.05, and different small letters represent statistical differences.

Memory function and spatial learning were measured according to Y-maze test (Figure 13). In the number of arm entries representing the mobility of the mouse, there was no significant difference between all groups (Figure 13a). The alternation behavior representing memory function and spatial learning of the SM (24.97%), NC (28.11%), and NS (26.53%) groups represented no significant differences. Alternation behavior (17.04%) of the PM group showed a significant decrease. However, the CF50 (23.37%) and CF100 (30.94%) groups showed improved alternation behavior (Figure 13b,c).

#### 2.13.2. Passive Avoidance Test

**Figure 14:**
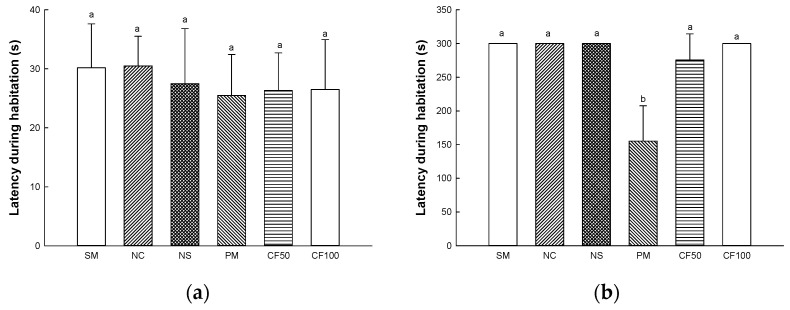
Protective effect of AECF on latency during habitation (**a**) and step-through latency (**b**) in passive avoidance test. Results shown are mean ± SD (*n* = 5). Data were statistically considered at *p* < 0.05, and different small letters represent statistical differences.

Short-term learning and memory function was evaluated according to the passive avoidance test (Figure 14). On the first day of the experiment, it was confirmed that there was no significant difference between all groups in delaying time to enter the dark zone (Figure 14a). On the second day of the experiment, in the result of the delay time to enter the dark zone, the SM (300 s), NC (300 s), and NS (300 s) groups represented no significant differences. The latency during habitation (155.16 s) of the PM group showed a significant decrease. However, the CF50 (275.67 s) and CF100 (300 s) groups showed improved memory dysfunction (Figure 14b).

#### 2.13.3. Morris Water Maze Test

**Figure 15:**
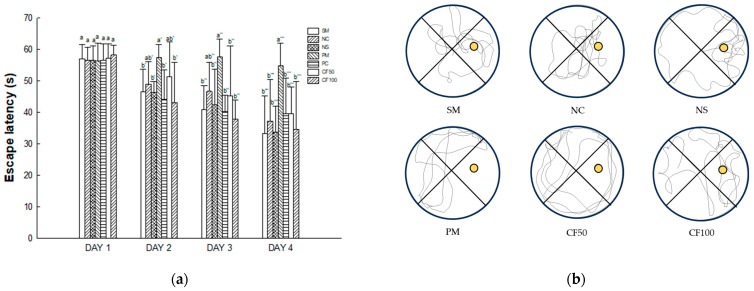
Protective effect of AECF on escape latency in hidden trial (**a**) and path tracing (**b**) in Morris water maze test. Results shown are mean ± SD (*n* = 5). Data were statistically considered at *p* < 0.05, and different small letters represent statistical differences. a-b means trials on first day. a’-b’ means trials on second day. a’’-b’’ means trials on third day. a’’’-b’’’ means trials on 4th day.

Long-term and learning memory capacity were evaluated according to the Morris water maze test (Figure 15). On the first day of the Morris water maze test, there were no significant differences in delay time to find the platform between groups in the SM (33.32 s), NC (37.28 s), and NS (33.85 s) groups on the fourth day of training. The escape latency (54.82 s) of the PM group showed a significantly increased delay time to find the platform. However, the CF50 (39.57 s) and CF100 (34.69 s) groups decreased the delay time to find the platform.

## 3. Discussion

PM_2.5_ is an air pollutant that causes damage to various organs when absorbed by the human body [20]. PM_2.5_ is known to induce oxidative stress, inflammation, and apoptosis and change the gut microbiome. According to a recent study, the deterioration of gut health and changes in the gut microbiome have been reported to affect brain function through the gut-brain axis [21]. Therefore, in this study, the protective effect of AECF on PM_2.5_-induced cognitive impairment was evaluated through the regulation of the gut–brain axis.

PM_2.5_ is produced by various sources such as dust, tire wear, construction work, and exhaust emissions and contains substances such as PAHs, transition metals, sulfates, and nitrates [22]. These PAHs activate the aryl hydrocarbon receptor (AhR) and promote the production of ROS and impairment of mitochondrial function in cells [23]. In addition, heavy metals such as lead, cadmium, and mercury contained in PM_2.5_ can deplete antioxidants and enzymes such as CAT, GSH, and SOD in the body and increase the production of ROS such as hydroxyl radicals and superoxide radicals, which causes oxidative stress [24,25]. Also, brain tissue is more vulnerable to oxidative stress because it is rich in unsaturated fatty acids and uses relatively more oxygen [26]. Therefore, the improvement effect of AECF on oxidative stress induced by PM_2.5_ was studied. In this study, decreases in antioxidant systems such as GSH and SOD and increased MDA in colon and brain tissues were evaluated. However, AECF ameliorated the antioxidant deficit by regulating the SOD, reducing GSH and MDA contents (Figure 2). In addition, AECF significantly reduced MPO, promoting the production of ROS and RNS to regulate the inflammation-related signaling pathways (Figure 3) [27]. In a previous study, AECF showed protection of the HT22 and BV-2 cells by increasing the cell viability and decreasing ROS production against PM_2.5_-induced cytotoxicity [14]. It has been reported that *Codium fragile* contains various compounds with physiological activities such as gallic acid, 4-hydroxbenzaldehyde, 4-hydroxybenzoic acid, *p*-coumaric acid, and salicylic acid [28]. Gallic acid significantly improved the level of antioxidant enzymes in the body such as CAT, SOD, and glutathione peroxidase and decreased the content of MDA [29]. According to Zhang et. al., *p*-coumaric acid showed lipid peroxide inhibitory activity that inhibited the oxidation of LDL by removing ROS [30]. Stearidonic acid contained in *Codium fragile* protected rat hippocampal cells from amyloid beta-induced oxidative stress by increasing total antioxidant capacity and mRNA expression of the catalase gene [31]. Sulfated polysaccharides isolated from *Codium fragile* effectively inhibited oxidative stress induced by H_2_O_2_ in in vitro and in vivo experiments [32]. Furthermore, phosphatidylcholine included in *Codium fragile* promotes the activities of enzymes such as adenylate cyclase, glutathione reductase, and Na-K ATPase and is known as a precursor of eicosanoids, prostaglandins, and other antioxidants [33]. Therefore, this study suggests that the aqueous extract of *Codium fragile* with physiological compounds might be an effective antioxidant against oxidative stress induced by PM_2.5_ inhalation.

The gut microbiome is known to be heavily involved in health and disease [34]. PM_2.5_ moves to the gut through mucociliary clearance transport or direct intake [6]. Various oxidative materials, including heavy metals, contained in PM cause disruption in protein synthesis and dysfunction of various enzyme systems, which affect the growth of beneficial bacteria [35]. The gut microbiome is related to various functions such as polysaccharide digestion, immunity, vitamin synthesis, angiogenesis, and behavioral function [7]. Absorbed PM causes an imbalance and changes of abundance in the gut microbiome and environment, such as changes in SCFAs and hormonal metabolism [36,37]. PM_2.5_ decreased beneficial bacteria such as *Lactobacillus*, *Lactococcus*, and *Lachnospiraceae*, which produce SCFA in intestinal tissues, and increased harmful bacteria such as *Bacillus* and *Escherichia*, which are related to gut disease and inflammation [26,36]. In particular, SCFA, a fatty acid containing six or less carbons, improves the gut environment to activate various receptors and regulate inflammation, tumors, energy metabolism, gut pH for the growth of beneficial bacteria, and oxidative stress by inhibiting histone deacetylases [38]. Intake of SCFAs maintains the integrity of tightly composed transmembrane proteins including claudins, occludin and the scaffolding protein ZO [39]. Tight junctions form a selective permeability barrier to restrict the intermixing and free diffusion of molecules through the intercellular space, apical and basolateral plasma membrane domains [40]. Tight junction protein plays a role in tightly binding gut epithelial cells and adjacent cells, but exposure to PM_2.5_ causes systemic diseases by releasing toxins and inflammatory cytokines decreasing tight junction [41]. Therefore, decreased SCFA and increased gut permeability due to PM_2.5_-stimulated change in the gut microbiome can induce systemic inflammation in the whole body [38]. It is necessary to maintain the gut environment by preserving the microbiome. In this study, the abundance of gut microbiome and SCFAs were measured to determine the effect of sulfated polysaccharide-rich AECF on gut health (Figure 4 and Figure 5). Inhalation of PM_2.5_ changed the gut microbiome, which reduced the SCFA content and caused loss of tight junctions, but AECF improved it. At the species level, an AECF diet increased *Firmicutes* and decreased *Bacteroides* in mouse feces. In results at the genus level, the abundance of *Alistipes, Lachnospiraceae* NK4A136 group, *Oscillospiraceae* NK4A214 group, and *Anaeroplasma* were decreased in the PM_2.5_ group. However, the administration of AECF improved the abundance of the genus level in the CF100 group. In addition, PM_2.5_ decreased tight junction proteins such as claudin-1 and occludin and shortened gut length, and AECF improved it (Figure 6). *Alistipes* and *Lachnospiraceae* were known to produce SCFAs with anti-inflammatory mechanisms [42]. In another study, the abundance of *Oscillibacter* and SCFA concentration were increased in mice fed polysaccharide, and similar results were shown in this study by increasing *Oscillibacter* and SCFAs [43,44]. *Desulfovibrio vulgaris* is a significant acetate producer that helps reduce systemic inflammation, apoptosis, and oxidative stress [45]. Phosphatidylchoine contained in *Codium fragile* improved the abundance of *Rikenellaceae* and *Lacnospiraceae* to improve the gut barrier and increased the production of gut SCFA [46]. Dietary polysaccharides such as galactan can be used in the gut by *Lactobacillus* and *Bifidobacteria* and improve the gut environment [47,48]. Similar to this study, *Porphyra tenera* extracts rich in sulfated galactan improved beneficial gut microbiome growth and the levels of tight junction protein expression such as occludin and claudin-1 [36]. In the aspect of the gut–brain axis, since the gut microbiome affects neuronal and myelin plasticity, and dysbiosis causes brain dysfunction such as cognitive dysfunction, improvement of the gut microbiome with prebiotics can improve learning ability and memory [49]. In addition, SCFA produced by the gut microbiome can improve cognitive dysfunction by enhancing the blood–brain barrier (BBB), regulating the development and function of microglia, improving the brain environment with the regulation of inflammation and neurotransmitters, and preventing gut leaking [41,50]. These results consider that AECF, which is rich in polysaccharides, improved PM_2.5_-induced dysbiosis, SCFA content, and expression of tight junction proteins. In conclusion, it was suggested that AECF might be a potential material for prebiotics that can improve the gut environment regulating the gut–brain axis.

Absorbed PM_2.5_ can be an inflammatory stimulator in the body [51]. PM_2.5_ binds to TLR-4 in cellular membranes and stimulates the MyD88 activating mitogen-activated protein kinase (MAPK) and NF-κB pathway [4]. IκB kinase is activated by intracellular signals such as TLR/MyD88 pathway phosphorylates IκB-α, resulting in ubiquitination of IκB-α and detachment from NF-κB, and moves into the nucleus to act as an inflammatory transcription factor [52,53]. When NF-κB moves into the nucleus, it generates pro-inflammatory cytokines such as TNF-a, IL-1β, monocyte chemoattractant protein-1, and vascular cell adhesion protein 1 [54]. In addition, ROS produced from PM_2.5_ induces the production of inflammasome, and this releases Cas-1 and mature pro-IL-1β [55,56]. Overexpressed inflammatory cytokines deteriorate the gut environment by causing intestinal dysfunction such as inflammatory bowel disease [20,26]. Additionally, TLR-4/MyD88 inflammatory signals and oxidative stress are induced by PM_2.5_ phosphorylates JNK, extracellular signal-regulated kinase 1/2, and p38, and these signals continuously induce the imbalance of BAX/BCl-2, release cytochrome C, and activate caspase cascades related to apoptosis [57,58]. An increase in the apoptosis signal causes mitochondrial dysfunction by deteriorating MMP and increasing mitochondrial ROS production [59]. Furthermore, continuous intestinal apoptosis decreases gut epithelium permeability and causes inflammation and abnormality of gut microbiome abundance [60]. In particular, the overactivation of the inflammatory response can affect brain function [11]. Inflammatory cytokines produced in intestinal tissues easily pass the BBB and induce neurodegenerative diseases such as AD and Huntington’s disease [61,62]. Therefore, this study was conducted to evaluate the improvement effect of AECF on PM_2.5_-induced inflammation and apoptosis related to cognitive dysfunction. PM_2.5_ activated various inflammation cytokines and apoptotic cascade protein expression via TLR-4/MyD88 pathways and disruption of the BAX/BCl-2 ratio, but the aqueous extract of *Codium fragile* improved them (Figure 7, Figure 8, Figure 9 and Figure 10). In a recent study, the aqueous extract of *Codium fragile* ameliorated osteoarthritis by regulating the MAPK/NF-κB pathway in IL-1β-induced osteoarthritis rats [63]. In a study by Han et al., the ethanolic extract of *Codium fragile* regulated inflammation by inhibiting JNK phosphorylation and mRNA expression of inducible nitric oxygen synthase (iNOS), COX-2, IL-6, nitric oxide (NO) and prostaglandin E2 (PGE-2) in lipopolysaccharide (LPS)-induced RAW 264.7 cells [64]. *Codium fragile* prevented apoptosis by inhibiting the protein expression of COX-2, iNOS, and TNF-α in UVB-treated HaCaT cells and BALB/c mice [17]. In addition, inflammatory cytokines such as IL-1β, TNF-α, and IL-8 were reduced when sulfated polysaccharides were injected into rock fish infected with fish bacteria [65]. The treatment of sulfated polysaccharides isolated from *Codium fragile* improved cell viability and decreased the production of NO and PGE-2 in LPS-induced RAW264.7 cells [66]. Polyunsaturated fatty acid reduced IL-1, IL-6, and TNF-α to prevent inflammation [67]. α-linolenic acid protein recovered the BCl-2/BAX ratio and expression of Cas-1 in doxorubicin (DOX)-induced hearts [68]. Furthermore, oleamide contained in *Codium fragile* inhibited the activation of NF-κB by inhibiting the production of NO and PGE-2 and protein expression of iNOS and COX-2 in LPS-treated BV-2 cells [69]. In addition, sulfated polysaccharides protected LLC-PK1 cells from cyclosporine-A-induced apoptosis by regulating the expression level of cytochrome C and Cas-3 [70]. Oleamide ameliorated apoptosis by inhibiting the activity of Cas-3 in rat cerebellar granule neurons cultured with K^+^ deficiency [71]. The tocopherol contained in *Codium fragile* was shown to protect endothelial cells from oxidized LDL by inhibiting increased ROS production and activating the expression of Cas-3 [72,73]. Furthermore, *p*-coumaric acid, one of the phenolic compounds in *Codium fragile,* inhibited apoptosis by suppressing the overexpression of BAX and Cas-3 and improving the expression level of BCl-2 in ethanol-induced mice kidneys [74]. Therefore, it is suggested that AECF might be a potential material to prevent PM_2.5_-induced cytotoxicity, improving inflammation and apoptosis to form a healthy gut environment and prevent cognitive dysfunction.

PM_2.5_ increase the production of ROS in mitochondria in the central nervous system (CNS) due to peripheral neuronal immune responses, increasing oxidative stress [75]. Damage to the mitochondrial membrane by PM_2.5_-induced oxidative stress causes mitochondrial dysfunction such as mitochondrial electron transport chain enzymes, mitochondrial respiration, and oxidative phosphorylation [76,77]. Dysfunction of the mitochondria disrupts ion homeostasis and leads to energy metabolism disorders [78]. Ultimately, PM_2.5_-induced ROS reduces the MMP level and induces damage to cellular function and apoptosis, causing cognitive dysfunction [79]. In this study, exposure to PM_2.5_ caused mitochondrial dysfunction by inducing ROS production and abnormal MMP levels. However, the consumption of AECF improved the mitochondrial deficits (Figure 11). Oleamide improved cell viability and mitochondrial function in 3-NP-induced rat cortical slices [80]. Unsaturated fatty acids contained in *Codium fragile* prevented calcium ion-induced mitochondrial permeability transition pore opening to protect the mitochondria by inhibiting mitochondrial depolarization, loss of ATP, and releases of cytochrome C [81]. In addition, SCFA produced in the gut microbiome restored damaged MMP and reduced calcium control disorders and mitochondrial ROS in brain cells [82]. Furthermore, the supplement of SCFAs protected mitochondrial damage by improving the function of mitochondrial respiration and protein expression of mitochondrial fission related to mitochondrial energy metabolism and radical production [83]. Therefore, there is a need to protect the mitochondria by intaking various fatty acids and SCFAs. In this study, it was confirmed that AECF effectively helped to protect brain mitochondria from PM-induced damage by reducing ROS and increasing MMP.

PM_2.5_-inducecd inflammation, apoptosis, and oxidative stress can damage the cholinergic system related to cognitive function in the CNS [84]. PM_2.5_ can move to the brain due to its small diameter, and PM_2.5_ that reaches the brain causes oxidative stress and inflammation [85]. In addition, PM_2.5_ contains heavy metals, nitrates, and sulfates and combines with the choline system that regulates the neural transmission system of these brain tissues and changes proteins and enzymes, which causes cellular dysfunction such as neuronal apoptosis, inflammation, and cognitive dysfunction [20]. In addition, PM_2.5_ overexpresses AChE and reduces the ACh contents, causing cognitive dysfunction and memory loss [86]. Similar to these studies, PM_2.5_ damaged the cerebral cholinergic system. However, the intake of AECF improved the damage to the cholinergic system by regulating ACh contents, ACh activity, and protein expression levels of AChE and ChAT (Figure 12). According to Ahn et al. (2021), *Codium fragile* extract contains various lysophosphatidylcholines that directly affect ACh synthesis in brain tissue and maintain membrane synaptic function [72,87]. Acetate is absorbed by nerve terminals and converted to acetyl-CoA, thereby promoting the formation of ACh and its release into the synapse [88]. Gallic acid, one of the physiological compounds of *Codium fragile*, reduced the activity of AChE in ketamine-induced psychosis mice [89]. Additionally, hydroxybenzoic acid inhibited the hydrolysis of ACh by inhibiting the activity of AChE [90]. Therefore, it is suggested that AECF containing various physiological compounds such as polyunsaturated fatty acids might be used for functional materials to ameliorate PM_2.5_-induced cholinergic system abnormalities and cognitive dysfunction.

The gut and brain are connected through two-way communication called the gut–brain axis [12,91]. Gut microbiome balance and the gut health of hosts have been found to affect the brain through the gut–brain axis [92]. The gut microbiome releases cytokines and inflammatory metabolites and microbial-derived molecules in the CNS and increases blood–brain barrier permeability through systemic circulation [93]. Gut-releasing substances and PM_2.5_ contained in the bloodstream can pass through the reduced permeability of the BBB and reach the brain [6,94]. After PM_2.5_ reaches the brain, cognitive dysfunction can occur due to TLR-4/MyD88-mediated inflammation, apoptosis, mitochondrial dysfunction, and cholinergic system disorders [20]. Therefore, this study confirmed the protective effect of the aqueous extract of *Codium fragile* on PM_2.5_-induced cognitive impairment via in vivo tests such as the Y-maze, Morris water maze, and passive avoidance tests by regulating the gut–brain axis (Figure 13, Figure 14 and Figure 15). In this study, cognitive dysfunction appeared in mice exposed to PM, and AECF intake restored cognitive function. *p*-coumaric acid, a physiological compound in AECF, improved cognitive dysfunction by alleviating hippocampal synaptic plasticity in aluminum chloride-induced AD mice [95]. The administration of oleamide improved scopolamine-induced cognitive dysfunction [96]. Polyunsaturated fatty acid in *Codium fragile* is an excellent nutritional source for the improvement of cognitive functions [97,98,99,100]. In addition, in the above results, the cognitive function improvement mechanism of AECF was confirmed with the improvement of the gut-brain axis. Finally, in the previous results, AECF improved the impairment of the antioxidant system, inflammation, apoptosis, mitochondrial function, and cholinergic system with improvement of the gut microbiome, SCFA concentration, and tight junction related to the biomarkers of the gut-brain axis. In conclusion, AECF, which can regulate PM_2.5_-induced cognitive dysfunction via various pathways, might be a potential material for use in functional food or pharmaceutics.

## 4. Materials and Methods

### 4.1. Chemicals

Phenol, sulfuric acid, hydrochloride, trichloroacetic acid, barium chloride, dimethyl sulfoxide, gelatin, barium chloride, metaphosphoric acid, *o*-phthaldialdehyde, sodium hydroxide, phosphoric acid, thiobarbituric acid, mannitol, sucrose, HEPES sodium salt, EGTA, potassium chloride, potassium phosphate, HEPES, 2′,7′-dichlorofluorescein diacetate (DCF-DA), pyruvate, tetrachloro-1,1,3,3-thtraethylbenzimidazolycarbo-cyanine iodide (JC-1), ferric chloride, bovine serum albumin, protease inhibitor, polyvinylidene difluoride (PVDF) membrane, and other solvents were obtained from Sigma-Aldrich Chemical Corp. (St. Louis, MO, USA). A superoxide dismutase (SOD) determination kit was purchased from Dojindo Molecular Technologies (Kumamoto, Japan). PM_2.5_ (mean diameter: 1.06 μm) was purchased from Power Technology INC. (Arizona Test Dust, Arden Hills, MN, USA).

### 4.2. Sample Preparation

The *Codium fragile* used in this study was purchased from Yeosu-si (Republic of Korea) in February 2018. To remove salt and impurities, it was washed until the salt concentration reached 0% and lyophilized with a vacuum drier (Operon, Gimpo, Republic of Korea). The lyophilized *Codium fragile* was extracted with 50-fold distilled water at 40 °C and filtered with No. 2 filter (Whatman Inc., Kent, UK). The extracted *Codium fragile* was concentrated using a vacuum rotary evaporator (N-N series, Eyela Co., Tokyo, Japan), and the re-lyophilized sample was stored at −20 °C until used.

### 4.3. Bioactive Compound Analysis

#### 4.3.1. UPLC Q-TOF MS^E^ Analysis

The AECF was dissolved with distilled water and fracted with hexane. The bioactive compounds of the AECF hexane layer were identified using the Waters Acquity ultra-performance liquid chromatography-quadrupole time of flight mass spectrometry (UPLC-QTOF/MS^E^) system (UPLC-Q-TOF/MS^E^, Xevo G2-S, Waters Corp., Milford, MA, USA). The physiological compounds were separated using the Acquity UPLC BEH C_18_ column (2.1 × 100 mm, 1.7 μm pore, Waters Corp, Milford, MA, USA) in positive ion mode. The following solvent gradients (mobile phase A: distilled water with 0.1% formic acid and B: acetonitrile) were added as follows: 0 to 12.0 min, 0% to 80% B. The MS analyses conditions were set as follows: drying gas (N_2_) temperature 120 °C, nebulizer pressure 40 psi, drying gas flow 30 L/h, capillary voltage 3 kV, fragmented voltage 175 V, and mass range from 100 to 1500 *m/z* [36].

#### 4.3.2. Total Polysaccharide Contents

Lyophilized aqueous extract of *Codium fragile* was reacted with 5% phenol and 95% sulfuric acid at room temperature for 20 min. The absorbance was then measured at 490 nm using a microplate reader (Epoch 2, Winooski, VT, USA) [101]. The standard curve was calculated using glucose.

#### 4.3.3. Total Sulfate Contents

Lyophilized aqueous extract of *Codium fragile* and 1 M HCl were mixed and heated at 105 °C for 5 h. After that, it was reacted with 3% trichloro acetic acid and BaCl_2_-gelatin solution at room temperature for 20 min. The absorbance was then measured at 360 nm using a microplate reader (Epoch 2) [102]. The standard curve was calculated using K_2_SO_4_.

#### 4.3.4. Monosaccharide Composition

The monosaccharide composition of the aqueous extract of *Codium fragile* was determined using a high-performance anion-exchange chromatography with a pulsed amperometric detection (HPAEC-PAD) system (Dionex, Sunnyvale, CA, USA) using a CarboPacTM PA1 column (25 cm × 4 cm) with 18 mM NaOH for 15 min [103].

### 4.4. In Vivo Mouse Experimental Design

Samtako (Osan, Republic of Korea) presented six-week-old BALB/c male mice. The mice were randomly split into four cages and bred in controlled standard laboratory conditions with 55% humidity, 22 ± 2 °C in 12 h light/dark cycle. Experimental mice were divided into 7 groups (*n* = 20; 10 for in vivo and ex vivo tests; 5 for the mitochondrial test; 5 for Western blot analysis) as a sham control (SM) group (without chamber exposure), a normal control (NC) group (with clean air exposure), a normal sample group (with clean air exposure and AECF treatment group (100 mg/kg of body weight), a PM group (with PM_2.5_ exposure), and a CF 50 group and a CF 100 group (with PM_2.5_ exposure and 50 and 100 mg/kg of body weight, respectively). The AECF was dissolved in drinking water and was fed orally with a stomach tube as a zonde needle for 12 weeks once a day. The experimental mice were exposed to PM_2.5_ at a 500 μg/m^3^ concentration in the whole-body exposure chamber for 5 h per day for 12 weeks. All animal experimental protocols were carried out under the Institutional Animal Care and Use Committee (IACUC) of Gyeongsang National University (certificate: GNU-210803-M0069, approved on 3 August 2021) with the approval of the Ethics Committee of Ministry of Health and Welfare, Republic of Korea.

### 4.5. Tissue Preparation

The mice were sacrificed to take colon and brain tissues. The tissues were homogenized in phosphate-buffer saline (PBS buffer, pH 7.4) using a bullet blender (Next Advance Inc., Averill Park, NY, USA) for ACh contents, AChE activity, SOD contents, and MDA contents, and 10 mM phosphate buffer (pH 6.0) for reduced GSH contents. The protein contents of homogenized tissues were measured using the Bradford protein assay [104].

### 4.6. Antioxidant System

#### 4.6.1. SOD Contents

To measure the SOD contents, after centrifuging the homogenized colon and brain tissue in PBS buffer at 4 °C for 10 min under 400× *g*, pellets without supernatants were used. After the pellets and 1 × cell extraction buffer (10% SOD buffer, 0.4% (*v*/*v*) triton X-100, and 200 μM phenylmethan sulfonyl fluoride) were centrifuged at 4 °C for 10 min at 10,000× *g*. After processing, SOD contents were measured on absorbance 450 nm (Epoch 2) using the commercial SOD kit (Dojindo Molecular Technologies) [5].

#### 4.6.2. Reduced GSH Contents

To measure the reduced GSH contents, the homogenized colon and brain tissue were centrifuged in PBS buffer at 4 °C for 15 min under 10,000× *g*. The supernatants were reacted with 5% metaphosphoric acid and re-centrifuged at 2000× *g*. The supernatants were reacted with 0.65 N NaOH, 0.26 M of tris-HCl (pH 7.8), and 1 mg/mL of o-phthaldialdehyde at room temperature for 15 min. Then, the fluorescence was measured at an excited wavelength of 320 nm and an emission wavelength of 420 nm using a fluorescence microplate reader (Infinite 200, Tecan Co., Mannedorf, Switzerland) [105].

#### 4.6.3. MDA Contents

To measure the MDA contents, homogenized colon and brain tissue were centrifuged in PBS buffer at 4 °C for 10 min under 10,000× *g*. The supernatants were then reacted with 0.67% thiobarbituric acid and 1% phosphoric acid at 95 °C for 1 h. After centrifuging the reactants for 10 min under 600× *g*, the supernatants were measured at 532 nm [106].

#### 4.6.4. MPO Activity in Colon Tissues

To measure the MPO activity, colon tissue was homogenized with 0.5% hexadecyltrimethylammonium bromide (HTAB) in 50 mM phosphate buffer (pH 6.0) and centrifuged at 15,000× *g* at 4 °C for 15 min. The supernatants and 50 mM potassium phosphate buffer (pH 6.0) containing o-dianisidine dihydrochloride (TCI, Tokyo, Japan) and 0.0005% hydrogen peroxide were mixed. The absorbance was detected at 450 nm with a microplate reader (Epoch 2). MPO activity was detected in units (U) of MPO/mg tissue, considering that 1 unit was defined as the amount required to disassemble 1 μmol of peroxide/min [26].

### 4.7. Western Blot

The colon and brain tissues were homogenized for 10 min in lysis buffer (GeneAll Biotechnology, Seoul, Republic of Korea) with a 1% protease inhibitor and centrifuged at 4 °C for 15 min under 13,000× *g*. The protein of the tissue was separated by SDS-PAGE gel and transferred to PVDF membrane. The transferred membrane was reacted with the primary antibody at 4 °C for 12 h and reacted with the secondary antibody at room temperature for 1 h [107]. The produced antibody complex was reacted with an ECL solution (TransLab, Daejeon, Republic of Korea) and colored, and detected using Chemidoc (iBright^tm^ CL1500 instrument, Invitrogen, Carlsbad, CA, USA). The density of the band was calculated using ImageJ software (ver. 13.0.6., National Institutes of Health, Bethesda, MD, USA).

### 4.8. 16S rRNA Sequencing

The mice feces were obtained and stored at −70 °C until use. 16S rRNA sequencing was performed using an Illumina Miseq platform by Sanigen Co., Ltd. (Anyang, Republic of Korea). rRNA amplifying was conducted using 16S rRNA V3-V4 region targeting primers through polymerase chain reaction (PCR) (T100 Thermal Cycler, Bio-Rad, Hercules, CA, USA). The PCR condition was set as follows: initial denaturation at 95 °C for 3 min, followed by 25 cycles of denaturing at 95 °C for 30 s, annealing at 55 °C for 30 s, elongation at 72° C for 30 s, and a final extension at 72 °C for 5 min [26].

Paired-end Miseq Illumina reads (2 × 300 bp) were processed using QIIME2 (version 2020.08), and the quality of the raw data was confirmed using FastQC (ver. 0.11.8) by Babraham Institute (United Kingdom). After quality inspection, adapters, primers, and noise were removed. Then, the sequence data were identified down to the species level using the Sliva 16S rRNA database [26].

### 4.9. SCFA Concentartion Analysis

The feces were homogenized in 5 Mm NaOH and centrifuged at 12,000× *g* for 10 min. The supernatant was mixed with propanol/pyridine (*v*/*v* = 3:2) solvent and propyl chloroformate to derivatize. The mixture was mixed with hexane and centrifuged at 15,000× *g* for 10 min. The hexane extraction was used to evaluate the SCFAs concentration using an Agilent 7890A Gas Chromatograph (Agilent, Santa Clara, CA, USA) and a DB-5MS column (thickness, 0.25 μM; length, 30 m; diameter, 0.25 mm, Agilent). The conditions were set as follows: split ratio, 50:1; injection temperature, 260 °C; column oven temp, 40 °C; flow rate, 1.0 mL/min. Helium was used as the carrier gas, and the total program was over at 16.5 min [26].

### 4.10. Mitochondrial Activity

#### 4.10.1. Mitochondrial Isolation

Brain tissue was homogenized with 10-fold volumes of mitochondrial extraction solution (75 mM sucrose, 0.1% BSA, 215 mM mannitol, and 20 mM HEPES sodium salt, pH 7.2) containing 1 mM EGTA. The homogenized tissues were centrifuged at 4 °C for 10 min under 1300× *g*, and the obtained supernatants were centrifuged again at 4 °C for 10 min under 13,000× *g*. After the supernatant was removed, pellets were mixed with a mitochondrial extraction buffer containing 0.1% digitonin. After 5 min, the mixture was reacted with a mitochondrial extraction buffer containing 1 mM EGTA and centrifuged at 4 °C for 15 min under 13,000× *g* [108].

#### 4.10.2. Mitochondrial ROS Contents

The mitochondria separated by the above process were reacted with KCl-based respiration buffer [2 mM potassium phosphate monobasic, 125 mM potassium chloride, 20 mM HEPES, 500 μM EGTA, 1 mM magnesium chloride, 2.5 mM malate, and 5 mM pyruvate] containing 25 μM DCF-DA. DCF fluorescence was measured at an excited wavelength of 485 nm and an emission wavelength of 530 nm using a fluorescence microplate reader (Infinite 200) [108].

#### 4.10.3. MMP Levels

The mitochondria separated from the mitochondrial isolating buffer containing 5 mM pyruvate and 5 mM malate were reacted with 1 μM JC-1 at a 96-well black plate. The mixture was incubated under darkroom conditions for 20 min, and then the fluorescence was measured at an excited wavelength of 530 nm and an emission wavelength of 590 nm using a fluorescence microplate reader (Infinite 200) [108].

### 4.11. Cholinergic System Test

#### 4.11.1. ACh Contents

For ACh contents, after homogenizing brain tissues with 10-fold volumes of PBS buffer, they were centrifuged at 4 °C for 15 min under 12,000× *g*. The obtained supernatants were mixed with a hydroxylamine regent, including 0.1 M HCl and 3.5 N NaOH, and reacted at room temperature for 1 min. After the reaction, 0.5 N NaOH and 0.3 M ferric chloride were added, and then absorbance was measured at 540 nm using a microplate reader (Epoch 2) [109].

#### 4.11.2. AChE Activity

For AChE activity, the supernatant used in the ACh contents analysis was reacted with 50 mM PBS buffer at 37 °C for 10 min. After adding Ellman’s reaction mixture, absorbance was measured at 405 nm using a microplate reader (Epoch 2) [110].

### 4.12. Behavial Test

#### 4.12.1. Y-Maze Test

The Y-maze is a black plastic maze with 33 cm (length) × 15 cm (height) × 10 cm (width) and is divided into three zones. After each area was divided into A, B, and C, the mouse was placed in one section, and the movement path of the mouse was measured by the video tracking system (SMART v3.0, Panlab SL, Barcelona, Spain) for 8 min [111].

#### 4.12.2. Passive Avoidance Test

The passive avoidance test was conducted in a chamber consisting of a dark side without lighting and a bright side with lighting, and the chamber had a passage with the door so that experimental mice could move each side. The mice were adapted without turning on the light for 1 min in the bright side, and after the light was turned on, the mice were adapted for 2 min. After that, when the passage was opened, and the mouse moved to the dark side, an electrical shock (0.5 mA, 3 s) was applied, and then the time of the first latency was recorded. After 24 h, the step-through latency time to re-enter the dark side was recorded (maximum time: 300 s) [112].

#### 4.12.3. Morris Water Maze Test

The Morris water maze test was conducted with a circular steel pool (90 cm in diameter and 30 cm deep) filled with water at 23 ± 2 °C and a height of 60 cm. The pool was randomly divided into quadrants (N, S, E, and W). The submerged platform was located in the center of the W quadrant. The platform was submerged at 1 cm underwater for invisibility, and the mice were randomly placed on one of the quadrants. The mice repeatedly swam four times a day for four days. When the mice found the submerged platform within 60 s, they were allowed to stay on the platform for 15 s. In case they hadn’t found the platform, the mice were trained to stay on the platform for 20 s. The escape latency was recorded using the video tracking system (SMART v3.0) for 60 s [113].

### 4.13. Statistical Analysis

All experimental results were presented as mean ± standard deviation (SD). The statistical significance of differences among groups was represented by different small letters and calculated by one-way analysis of variance (ANOVA). Means with different letters presented are significantly different. Duncan’s new multiple-range test (*p* < 0.05) with SAS ver. 9.4 (SAS Institute Inc., Cary, NC, USA) determined significant differences.

## 5. Conclusions

In this study, the aqueous extract of *Codium fragile* (AECF) was confirmed to evaluate an anti-amnesic effect by regulating systemic inflammation and gut–brain axis induced by PM_2.5_. AECF, which contained the rich sulfated polysaccharide, unsaturated fatty acid, and fatty acid amide, regulated the abundance of the gut microbiome, SCFA contents, and protein expression levels of tight junction in colon and brain tissues. The treatment of AECF protected the antioxidant system and regulated protein expression levels of inflammation and apoptosis against PM_2.5_ exposure in colon tissues. AECF enhanced PM_2.5_-induced cholinergic and mitochondrial dysfunction in brain tissues. Ultimately, the consumption of AECF enhanced cognitive dysfunction by regulation of the gut–brain axis via the TLR-4/MyD88 pathway. In conclusion, it suggests that AECF might be a potential marine resource that can prevent cognitive dysfunction by protecting the gut–brain axis against PM_2.5_-induced dysbiosis.

## Figures and Tables

**Table 1:** Compounds identified from aqueous extract of *Codium fragile* hexane layer.

No.	RT ^a^ (min)	Parent Ion ^b^ (*m/z*)	Fragment Ion (*m/z*)	Identified Compounds
1	6.62	277	107, 93, 91, 79, 67	Stearidonic acid
2	6.99	279	227, 157, 95, 81	Linolenic acid
3	7.79	254	237, 219, 149, 135	Palmitoleamide
4	7.99	280	263, 245, 175, 113, 95, 81	Linoleamide
5	8.36	256	116, 102, 88, 74, 57	Hexadecanamide
6	8.46	282	283, 265, 247, 240	Oleamide
7	8.95	284	285. 102, 88	Octadecanamide

^a^ RT means retention time. ^b^ Ions are presented at *m*/*z* [M+H]^+^.

**Table 2:** Total polysaccharide contents, sulfate contents, and free sugar contents of *Codium fragile*.

Total Polysaccharide (%)	Sulfate (%)	Relative Area (%)					
		Galactose	Arabinose	Glucose	Xylose	Fucose	Rhamnose
31.18 ± 1.20	31.05 ± 2.81	48.81 ± 0.51	21.35 ± 0.17	20.27 ± 0.02	7.84 ± 0.31	0.89 ± 0.04	0.80 ± 0.04

The results were presented as the mean ± SD (*n* = 3). Data were statistically considered at *p* < 0.05.

## Data Availability

The data underlying this article are shared upon reasonable request to the corresponding author.
